# Development of a Chitosan-Based Film from Shellfish Waste for the Preservation of Various Cheese Types during Storage

**DOI:** 10.3390/foods13132055

**Published:** 2024-06-27

**Authors:** Rossella Vadalà, Laura De Maria, Rita De Pasquale, Eleonora Di Salvo, Giovanna Lo Vecchio, Giuseppa Di Bella, Rosaria Costa, Nicola Cicero

**Affiliations:** 1 Dipartimento di Scienze Biomediche, Odontoiatriche e delle Immagini Morfologiche e Funzionali, University of Messina, 98168 Messina, Italy; rvadala@unime.it (R.V.); laura.demaria@studenti.unime.it (L.D.M.); eleonora.disalvo@unime.it (E.D.S.); vanialovecchio25@gmail.com (G.L.V.); giuseppa.dibella@unime.it (G.D.B.); ncicero@unime.it (N.C.); 2 Science4life S.r.l. Start Up, 98168 Messina, Italy; r.d.pasquale@hotmail.com

**Keywords:** film, chitosan, cheese, shrimp shell waste, packaging, sustainability

## Abstract

The global concern about the use of disposable plastics has fed the research on sustainable packaging for food products. Among the virtuous materials, chitosan emerges as a valid alternative to conventional polyethylene films because of its abundance in nature. In this work, a novel film for food wrapping was developed by exploiting shellfish waste according to a vision of circular economy. Compared to previous studies, here, novel ingredients, such as polyvinyl alcohol (PVA), fibroin, and essential oils, were used in a synergistic combination to functionally postpone cheese deterioration. The fermentative procedure applied for the obtainment of chitin contributes to filling the existing gap in the literature, since the majority of studies are based on the chemical pathways that dramatically impact the environment. After pretreatment, the shrimp shell waste (SSW) was fermented through two bacterial strains, namely *Lactobacillus plantarum* and *Bacillus subtilis*. A deacetylation step in an alkaline environment transformed chitin into chitosan, yielding 78.88 g/kg SWW. Four different film formulations were prepared, all containing chitosan with other ingredients added in order of decreasing complexity from the A to D groups. The novel films were tested with regard to their physico-mechanical and antioxidant properties, including the tensile strength (12.10–23.25 MPa), the elongation at break (27.91–46.12%), the hardness (52–71 Shore A), the film thickness (308–309 μm), and the radical scavenging activity (16.11–76.56%). The performance as a cling film was tested on two groups of cheese samples: the control (CTR), wrapped in conventional polyethylene (PE) film; treated (TRT), wrapped in the chitofilm formulation deemed best for its mechanical properties. The volatiles entrapped into the headspace were investigated by means of the SPME-GC technique. The results varied across soft, Camembert, and semi-hard cheeses, indicating a growing abundance of volatiles during the conservation of cheese. The bacterial growth trends for mesophilic, enterobacteriaceae, and lactic acid bacteria were expressed as the mean colony forming units (CFU)/mL for each type of cheese at different sampling times (day 2, day 8, and day 22): the highest load was quantified as 8.2 × 10^6^ CFU/mL at day 22 in the CTR Camembert cheese. The TRT samples generally exhibited inhibitory activity comparable to or lower than that observed in the CTR samples. The sensory analysis revealed distinctions in cheese taste between the TRT and CTR groups.

## 1. Introduction

In the last decades, there has been an increasing demand for sustainable packaging, mainly due to growing concerns about the widespread presence of plastics in the environment. As a matter of fact, a large amount of wasted plastic comes from food packaging, therefore raising attention from researchers on this issue. Biopackaging has emerged as a promising alternative to conventional plastic packaging, offering both time solutions and challenges in this respect. By definition, biopackaging consists of renewable resources, such as plant extracts and biodegradable plastics, which considerably reduce plastic waste and lower the carbon footprint [1]. Besides the benefits on the terrestrial ecosystem, biopackaging can provide practical advantages including an extended shelf life, improved food safety, and increased product visibility [2]. As a result, biopackaging is increasingly chosen by agri-food companies, also because it is safer for human health than traditional plastics (i.e., Styrofoam), classified as important contributors to cancer, infertility, and developmental disorders [3]. Pioneer biopackaging employs a variety of materials, such as cellulose, starch, whey, and soy [4].

About ten years ago, sustainable packaging technology started to explore a new type of material recovered mainly from shellfish waste: chitosan, a polymer derived from the deacetylation of chitin. The latter is the predominant component of the exoskeletons of crustaceans and insects [5]. The interest in chitosan has grown strongly, especially for the unique features that make it a potential and valid substitute for plastic films [6]; these include (i) biodegradability and sustainability (chitosan is derived from renewable sources and is degraded by enzymes into non-toxic substances) [7]; (ii) barrier properties against oxygen, water vapors, and other gases, exerting antispoilage and antioxidant effects; (iii) bioactivity (due to its chelating ability and cationic structure, chitosan exerts antimicrobial, antioxidant, and enzyme inhibition activities); (iv) mechanical properties that allow the obtainment of films with good flexibility and transparency; and (v) blending attitude (it can be combined with other polymers for the improvement of its physical properties).

Along with its several advantages, chitosan-based packaging presents challenges such as the cost of production, which entails higher prices compared to conventional plastics; the scalability of production; maintaining the desired properties over time; and low compatibility with some food types, in particular those presenting significant moisture and fat contents [8]. For these reasons, in the last years, new strategies in packaging technology have been developed by researchers in order to improve the mechanical and physical properties of this type of biopackaging [6]. Among these, those worth mentioning are (i) composite films, obtained by incorporating other natural polymers, such as starch, cellulose, or alginate; (ii) chitosan derivatization on the three active sites of the polymer, such as gallic acid–chitosan or quinoxaline-grafted chitosan; (iii) the addition of bioactives that improve the antimicrobial and antioxidant properties while maintaining unaltered sensory properties; (iv) chitosan nanoparticles that dramatically increase the contact surface with spoilage agents [6]. 

Despite the extensive research on the development and characterization of chitosan-based films for food applications documented by numerous studies proposing formulations with bioactive components, there are still several aspects that remain underexplored. The aim of the present study was the development and testing of a chitosan-based cling film with a novel formulation suitable for cheese preservation. Specifically, the study evaluated the synergistic action of chitosan and co-constituents—including a mix of essential oils from aromatic herbs, fibroin, prickly pear mucilage, and polyvinyl alcohol—in extending the shelf-life of various types of cheeses by postponing their microbiological deterioration, oxidative rancidity, and decline in nutritional and organoleptic values. 

The central points of the novelty and relevance of this work can be summarized as follows: (i) the functionalization of packaging for cheese wrapping; the literature presents considerable gaps on this issue despite other food categories, as confirmed in the recent review by Muñoz-Tebar et al. [9] (indeed, the microbial deterioration process of cheese exhibits a typical evolution strongly influenced by pH, water activity, nutritional composition, and the presence of bacteria intrinsic to the cheese’s metabolism) and; (ii) the sustainability of the film production process; also, in this case, a lack of works on the implementation of fermentative bioconversion techniques starting from shellfish waste is evident in the literature [9,10]. Many papers have been based on the employment of chitosan obtained through chemical methods, which negatively impacts the sustainability of the entire process. 

This study also highlights how the eco-friendly and effective pretreatment of raw materials positively influences the quality of the obtained chitosan (in terms of the deacetylation degree and molecular weight) and the fermentation yield. Here, we also introduce innovative ingredients into the film formulation, represented by fibroin and mucilage from prickly pear shovel, never utilized before in cling films [10,11,12]. Each co-constituent exerts a specific function: PVA acts as a waterproofing and elasticizing agent, also improving the mechanical performance of the wrapping (particularly the elongation at break) as well as its barrier properties against water vapor, oxygen, and carbon dioxide. Glycerol is used as a plasticizer owing to its non-toxicity, biodegradability, and compatibility with the film formulation. The incorporation of low molecular weight plasticizers such as glycerol in the film formulation increases the free volume of the matrix and enhances molecular mobility, significantly improving the mechanical strength. Moreover, polyols like glycerol contain hydrophilic groups that interact with chitosan through hydrogen bonds, leading to increased moisture absorption. The inclusion of nopal shows interesting plasticizing and antioxidant functions. Fibroin is a fibrous protein that can be isolated from the silk cocoons produced by the larvae of Bombyx mori. This protein is an attractive constituent for films, membranes, and coatings due to its property that enhances permeability to oxygen [13,14,15]. 

After preliminary microbiological and mechanical testing, the chitofilm was wrapped around some types of cheese to evaluate its performance as food packaging. Additionally, a sensory analysis was performed to evaluate the overall sensory attributes and consumer acceptance of the food products wrapped in the newly developed packaging. Finally, the volatile compounds entrapped into the headspace of wrapped cheese were monitored by means of solid-phase microextraction (SPME) and gas chromatography-mass spectrometry (GC-MS).

Therefore, this work focuses on the optimization of the functionality of chitosan-based films for food packaging in terms of the mechanical performance and ability to exert antioxidant and antimicrobial actions. Another aim of the study was to minimize the economic and environmental impacts of production, with a view towards future industrial applications of the method. In fact, when considering Europe, in 2020 alone, the production of polyethylene for packaging amounted to 13.5 million tons, with 23% of the total destined for the food sector [16]. On the other hand, FAO inferred an estimated production of 10 million tons of shellfish waste in 2021. Thus, it is easy to see how great the advantage for the industry can be to exploit this large amount of discarded materials, the disposal of which often represents a major problem for industrial producers.

## 2. Materials and Methods

### 2.1. Materials and Reagents

*Lactobacillus plantarum* (ATCC 14917) and *Bacillus subtilis* (ATCC 6633); peptone, meat extract; glucose and Man, Rogosa and Sharpe (MRS) broth were purchased from Oxoid-Unipath LTD (Basingstoke, Hampshire, UK); sodium chloride and DPPH (1,1-diphenyl-2-picrylhydrazyl) were provided by Sigma-Aldrich (Chemie, Darmstadt, Germany). Polyvinyl alcohol (PVA, Mw = 124,000–186,000 g.mol^−1^, 98–99% hydrolysis) was obtained from Aldrich Chemical Company Inc. (Milwaukee, WI, USA); acetic acid (1%, *v*/*v*; Sigma–Aldrich Chemie, Darmstadt, Germany); vegetable glycerol, 99.5% *v*/*v* and Tween 80 (polyethylene glycol sorbitan monolaurate) were purchased from Carlo Erba (Milan, Italy). The essential oils were obtained via steam distillation from the aerial parts of *Thymus vulgaris*; *Calamintha nepeta*; *Camellia sinensis*; *Origanum vulgaris*, were supplied by Esperis S.p.a. (Milano, Italy) and were stored at 4 °C until use. Fresh nopal (*Opuntia ficus*-*indica*) was purchased from Bioinagro Srl, (Licata, Agrigento, Italia); the fibroin solution (50 mg·mL^−1^) was supplied by Sigma-Aldrich (Chemie, Darmstadt, Germany). The shrimp shell waste (SSW) consisted of the exoskeletons, antennas, legs, eyes, rostrums, and tail edges of the species *Parapenaeus longirostris* coming from the 37.1 and 37.2 FAO zones. The wasted material was procured in the local fish markets of Messina (Italy).

### 2.2. Extraction of Chitin

*Lactobacillus plantarum* (ATCC 14917) was inoculated into a 250 mL flask containing 100 mL of MRS broth media and incubated at 37 °C for 24 h. The *Bacillus subtilis* (ATCC 6633) inoculum was prepared into a 250 mL flask containing 100 mL of Luria-Bertani (LB) media with peptone (5 g), meat extract (3 g), and NaCl (5 g) and incubated at 30 °C for 24 h using a shaking incubator (150× rpm). The prepared inocula yielded a cell concentration of approximately 10^8^ CFU/mL. At the same time, the SSW was pretreated using the following steps: first cleaning it with running water followed by distilled water; drying in a conventional heater at 90 °C for 3 h, and finally, grinding to a powder via a knife mill (Retsch Grindomix gm 200). The grain size was calculated by means of an optical granulometer (Haver Computerized Particle Analysis CPA 2-1), and estimated as around 1.2 mm. The pretreated SSW was stored at −20 °C until the fermentation tests. For the obtainment of chitin from the SSW powder, a batch fermentation was carried out, taking inspiration from previous work [17]. Fermentation took place in a 250 mL flask containing 100 mL of MRS broth supplemented with 80 g/L glucose and 5% (wt) of SSW. The culture medium was inoculated with 5% (*w*/*v*) of a combination of the two strains (1:1) and incubated at 30 °C for 160 h with shaking. The obtained chitin was refluxed with a 50% aqueous solution of NaOH at a temperature of 80 °C and shaking at 120 rpm; then, it was filtered and washed with distilled water and finally dried in a conventional heater at 60 °C for 12 h. The obtained chitosan was food grade, with an 85% deacetylation degree (DD) and 300,000 Da molecular weight (MW). The DD was estimated through NMR by means of a Bruker Avance 500 MHz instrumentation, with the spectrometer equipped with a smart probe. The MW was identified using viscometrical measurements according to the Mark–Houwink–Sakurada equations [18,19].

### 2.3. Film-Forming Solution and Deposition

**Figure 1:**
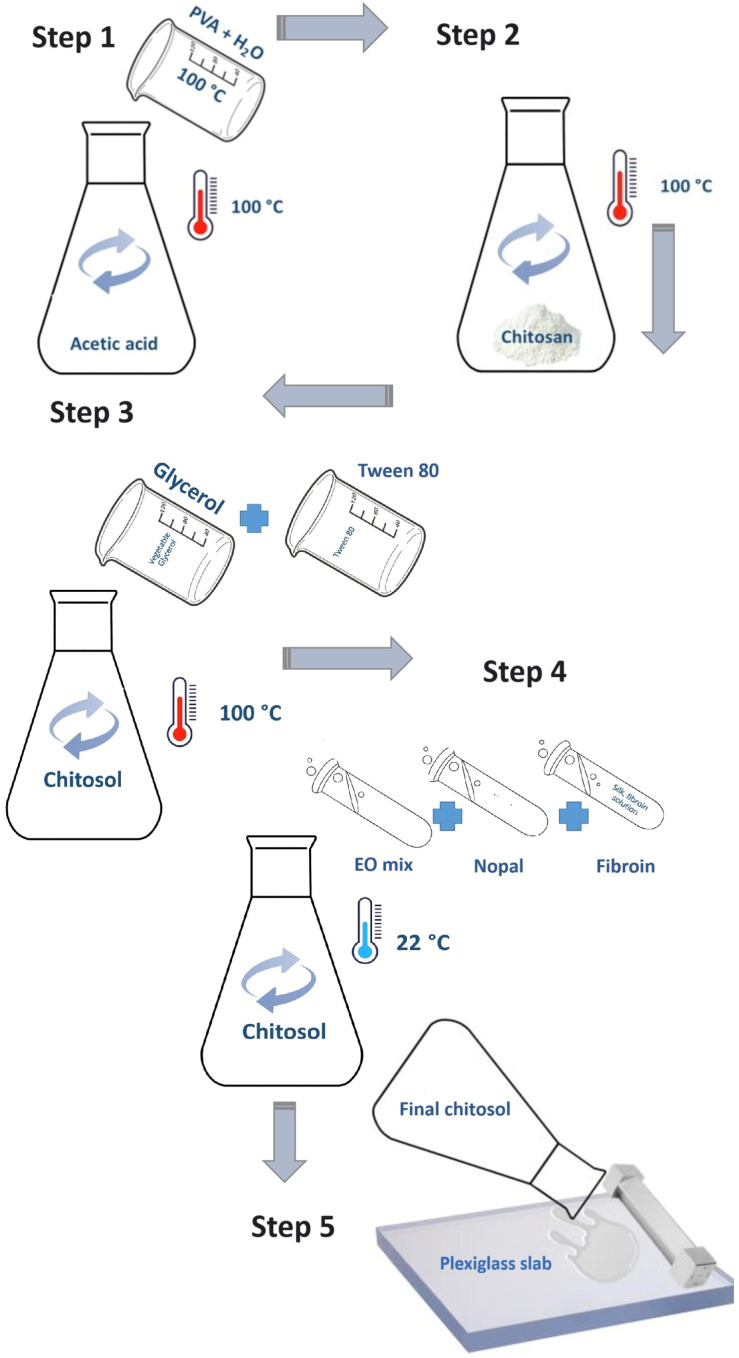
Scheme of the process implemented for the film preparation. PVA: polyvinyl alcohol; EO mix: essential oil mixture (*Thymus vulgaris*; *Calamintha nepeta*; *Camellia sinensis*; *and Origanum vulgaris*.); Nopal: mucilage from a shovel of prickly pear (*Opuntia ficus indica* L. Miller); fibroin: hydrolyzed silk protein.

The entire procedure of the film preparation is depicted through the scheme in Figure 1. Four different types of films were prepared, which were successively tested with respect to their mechanical features:

Group A: a complete formulation prepared as described in the current section;

Group B: a semi-complete formulation of the film-forming solution without polyvinyl alcohol; 

Group C: a partial formulation of the film-forming solution (chitosan, glycerol, and essential oil blend);

Group D: a basic formulation of the film-forming solution (chitosan and glycerol).

The films belonging to group A were prepared as follows. For the obtainment of 1 L of film, in a flask with stirring at a temperature of 100 °C and a rotation speed of 500 rpm, 3 g of granular polyvinyl alcohol (PVA) was added to 100 mL of distilled water until reaching the boiling point. PVA was added as a cross-linking agent capable of enhancing the gas barrier properties. It also improved the mechanical performance parameters of the final product, such as the elongation, resistance, elasticity, and film-forming attitude of the liquid formulation [13]. The dissolved PVA was added to a chitosan-based solution, which was prepared by dispersing it in a flask containing 20 g of chitosan in a 1% glacial acetic acid aqueous solution (780 mL) at 100 °C with stirring (500 rpm) for 10 min. The flask was placed on a magnetic plate at a temperature of 100 °C and at a rotation speed of 500 rpm until complete dissolution. As a natural plasticizer, 20 mL of vegetable glycerol (99.5% *v*/*v*) was added. Subsequently, the temperature was lowered to 80 °C for 5 min, and then 10 mL of Tween 80, as an emulsifier agent, was added to the solution. The film-forming solution was cooled down until its complete stabilization. Afterward, a mixture of essential oils was dispersed into the solution with stirring at 350 rpm for 5 min and at room temperature. The mixture consisted of 0.5 mL of each of the following oils: *Thymus vulgaris*; *Calamintha nepeta*; *Camellia sinensis*; and *Origanum vulgaris*. Finally, 4 mL of mucilage from a shovel of prickly pear (*Opuntia ficus indica* L. Miller) and 3 mL of a silk fibroin solution were added. The first is a natural thickener with strong antimicrobial and antioxidant activities [14], while the second improves the strength properties and water oxygen permeability [15]. The films were obtained via deposition and drying at 22 °C on a plexiglass slab of uniform layers (300 μm) of the film-forming solution. A chromium-plated steel film applicator (Wasag film applicator model 288, Erichsen GmbH & Co. KG, Hemer, Germany) was used to stretch the layers. The obtained films were conditioned in an environmental chamber at 23 °C and 52% relative humidity (RH) for 8 h according to the ASTM D618-13 [20].

### 2.4. Mechanical Testing

The obtained film was tested with respect to its suitability for food wrapping. In particular, mechanical properties, such as tensile strength and hardness, were evaluated in relation to the constituents of the film-forming solution [21]. Four groups of films, named A, B, C, and D, were assayed; each group contained five samples, and each sample was tested in triplicates. The mechanical properties of group A, obtained as described above in Section 2.3, were compared with those of the other groups of samples, which had semi-complete (group B), partial (group C), and basic (group D) formulations. The tensile strength (TS) and elongation at break (EB) were evaluated for each group of samples according to the international standard ASTM D882-18 [22]. The TS tests were carried out with the Universal Testing Machine Model 1000 (HIKS, Selfords, Redhill, England, UK). From each sample, three rectangular test pieces (size: length: 125 mm; width: 10 mm; thickness: ~0.3 mm) were obtained via die cutting. The thickness of each specimen was measured using the digital micrometer 49–56 from Messmer Büchel (Messmer Büchel-Industrial Physics, LLC, Veenendaal, The Netherlands). The environmental conditions of the TS tests were: a T of 23 ± 2 °C and relative humidity (RH) of 50 ± 10%, while the specimen conditioning consisted of 24 h at 23 ± 2 °C; RH of 50 ± 10%; a cross-head speed of 12.5 mm·min^−1^, and a distance between grips of 125 mm. The hardness was measured as Shore A, namely the measurement of the penetration resistance of a material by means of a penetrator with a preset force. The hardness was estimated for each group of samples according to the international standard UNI EN ISO 868:2005 [23]. Shore A was measured using the instrument H17 A (Wallace, Kingston, UK). The environmental conditions were a T of 23 ± 2 °C and RH of 50 ± 10%, while the specimen conditioning occurred for 24 h at 23 ± 2 °C and RH of 50 ± 10%.

### 2.5. DPPH Radical Scavenging Assay

An aliquot of 100 mg of film was immersed in 5 mL of ethanol (95%) and left to stand in the dark at 40 °C for three hours. A total of 1 mL of supernatant was added with 1 mL of a DPPH (1,1-diphenyl-2-picrylhydrazyl) solution, having a concentration of 0.06 mM in ethanol (95%). This mixture was kept in the dark with stirring for 30 min. The control specimen consisted of the sole DPPH solution. The absorbances of both the control and sample solutions were recorded in a UV–vis spectrophotometer (UV-2401PC, Shimadzu, Milan, Italy). The antioxidant activity was then measured according to the following equation [24]:

$$Antioxidant\;activity\;\%=\left[\frac{A_{control}-A_{sample}}{A_{control}}\right]\times 100$$

where:

*A_control_* is the control reaction absorbance;

*A_sample_* is the testing mixture absorbance;

Each group included 5 samples, each tested in triplicates. The results were expressed as the mean ± standard deviation.

### 2.6. Analysis of Wrapped Cheeses

#### 2.6.1. Samples

The cheese samples were purchased in dairy shops located in Messina (Italy) and were of the following types: soft cheese (48% humidity), Camembert (54% humidity), and semi-hard cheese (37% humidity). All samples were wrapped and grouped in two different batches: (i) the control (CTR), wrapped in commercial polyethylene film; and (ii) treated (TRT), wrapped in the group A film, also called chitofilm. The samples were kept refrigerated in a cabinet with a glass door at 5–6 °C until the analysis. The relative humidity of the cabinet was estimated to be 36 ± 1% by means of a digital thermo-hygrometer (TROTEC GmbH, Heinsberg, Germany), and it was measured in the cheese storage compartment. 

#### 2.6.2. Extraction of the Volatiles

The headspace solid-phase microextraction (HS-SPME) samplings took place on day 2, day 8, and day 22 after sample wrapping. On each sampling day, two samples (one from the CTR group and one from the TRT group) were extracted in triplicates for every type of cheese. The HS-SPME extraction was performed manually by piercing the film covering the cheeses. Preliminarily, the cheese samples (about 20 g each) were carved in such a way as to create a headspace in which the fiber could be easily exposed to volatile adsorption. The fiber coating was a DVB/Car-PDMS, which was 1 cm long and 80 μm thick (Agilent Technologies, Santa Clara, CA, USA). Presaturation occurred by thermostatting the samples for 10 min at 30 °C in an incubator. The fiber exposure lasted 20 min at 30 °C in an incubator, after which the analytes were desorbed for 5 min at 250 °C in the GC injector.

A Shimadzu GC-2010 system was used for the separation and quantification of the volatile analytes. The apparatus was equipped with a capillary column, namely an SLB-5ms (Merck, Darmstadt, Germany), which was 30 m × 0.25 mm with a 0.25 μm film thickness. Additionally, the oven temperature program started from 50 °C at 4 °C/min to 250 °C (2 min) at 10 °C/min to 300 °C (5 min). The injection occurred in the splitless mode; the sampling time was 5 min with a split ratio of 1:20, and the temperature was 250 °C. The carrier gas (He) linear velocity was 30 cm/s. The FID was 300 °C, and the gases were H_2_ (40 mL/min), N_2_ (80 mL/min), and air (400 mL/min). The data were handled by means of GCsolution 2.32 software.

The GC-MS analyses were performed on a Shimadzu GCMS-QP2010 equipped with the same capillary column as described above. The mass spectrometric parameters were as follows: source (EI): 200 °C, interface: 230 °C, scan speed: 10,000 amu/s, scan mass range: 35–350 *m*/*z*, mass spectral libraries: FFNSC 2 (Shimadzu, Kyoto, Japan), Adams 4th edition (Allured, Carol Stream, USA), Wiley 9 (John Wiley & Sons, Inc., Hoboken, USA), NIST17 (NIST, Gaithersburg, USA) and other homemade databases. The gas chromatographic runs were acquired at the same conditions as GC-FID. The data handling was achieved using GCMSsolution 2.5 software (Shimadzu, Kyoto, Japan). Besides library matching, single-peak identities were assigned according to the retention indices, which were measured in real samples after the SPME extraction and injection of the C8-C18 n-paraffin mixture [25].

#### 2.6.3. Microbiological Testing

In order to assess the performance of chitosan-based films as active packaging material, their impact on the microbiological quality and shelf-life of three distinct varieties of cheese that were wrapped with the chitofilm was estimated [26]. The microbiological testing aimed at defining the attitude of the film to enhance the shelf-life and to preserve the quality and safety of perishable food products, such as cheese. Three different groups of cheese samples were considered: soft cheese, Camembert, and semi-hard cheese. In each group, nine samples, considered as the controls (CTRs), were wrapped in conventional food-grade polyethylene film, and nine samples, considered as treated (TRT), were covered with the chitofilm. To simulate typical storage conditions, all samples were stored in a fridge at the temperature of 5 °C until testing. The microbiological quality and safety of the samples were investigated during storage. For each sample analyzed in triplicates, the growth of mesophilic (MES), enterobacteriaceae (ENT), and lactic acid bacteria (LAB) was detected on the 2nd, 8th, and 22nd days. The analysis of the mesophilic bacteria count was evaluated using a standard PCA at 30 °C for 72 h. The enterobacterial load was estimated on Violet Red Bile Glucose Agar after 48 h at 37 °C. Finally, the LAB load was counted on the MRSA at 37 °C after 48 h.

#### 2.6.4. Sensory Analysis

The sensory analysis was carried out in the PanLab laboratory of the Biomorf Department at the University of Messina. The panel was composed of six assessors chosen among expert personnel and graduate students aged 25–49 (mean = 35.3), with five females and one male. The panelists were previously trained with specific sessions in order to acquire the cheese flavor lexicon according to previous works [27,28]. To this aim, the assessors were trained to recognize the following attributes by means of reference standards: acidic (citric acid, natural yogurt); milky (heavy cream); buttery (diacetyl, unsalted butter); yeasty (blue cheese); herbal (powdered rosemary and thyme dry leaves); minty (peppermint essential oil); and savory (meat broth). The reference samples were assigned an intensity score of 6 over a 0–10 point scale (see the Supplementary Materials for the questionnaire). The soft, Camembert, and semi-hard cheese samples (wrapped 7 days before the panel test) from both the groups (CRT and TRT) were blinded and randomly provided. Each sample was assayed in duplicates with a 10 min break in between, during which the palates were rinsed with water. Before testing, the samples were removed from the film and equilibrated at an ambient temperature (20 ± 3 °C). The obtained data were subjected to statistical analyses (XLSTAT, Microsoft Excel, 2023).

### 2.7. Statistical Analysis

The dataset is expressed as the mean ± standard deviation (sd) of the triplicate measurements. Significant differences (*p* < 0.05) within the means were analyzed using a one-way ANOVA, then using Tukey’s honestly significant difference (HSD) test with the XLStat statistical software Microsoft Excel data analysis add-on (Microsoft Corporation, Redmond, WA, USA).

## 3. Results and Discussion

### 3.1. Mechanical Properties

The DD plays a fundamental role in the functions exerted by the chitosan polymer: as an example, the DD and the molecular weight of chitosan were the topic of investigation and were directly correlated with the antiadhesive and anticoagulant properties [29]. The final chitosan yield was 78.88 g kg^−1^ SSW (7.9% *w*/*w*), which is considerably lower than that generally achieved by using the chemical method of extraction: Naznin obtained values ranging from a 15 to 30% yield [30]. However, it is worthwhile emphasizing that the biological recovery of chitin from SSW via fermentation is definitely a greener technology; therefore, a lower yield represents an acceptable compromise. 

The results of the mechanical tests are presented in Table 1, where for each group (A, B, C, and D) of biodegradable films compared, the values of thickness (FT), tensile strength (TS), elongation at break (EB), and hardness (H), are expressed as the mean ± standard deviation. No significant differences (*p* ≥ 0.05) in terms of film thickness among the different groups tested were found. This uniformity in thickness is a positive finding, as it suggests that the manufacturing process is reliable across all the biodegradable film formulations. Group A showed the highest levels of tensile strength, elongation at break, and hardness, making it suitable for applications such as food wrapping. The higher mechanical performance of group A may be unambiguously attributed to the contribution of PVA. In fact, the presence of PVA leads to an average increase of 19.61% in TS, 8.52% in EB, and 29.09% in H when comparing groups A and B. Moreover, the EB estimated for group A reached a very significant value of 46.12%. This value, which is related to the ductility and elasticity of a material, was compared with those found in the literature for lab-made films with different formulations but thicknesses and testing conditions similar to our chitofilm.

According to Susmitha et al., 2021, a corn starch/gelatin-based food film reinforced with 5% of mango puree exhibited an EB of 35.4%, which is more than 10 points lower than that assessed for group A [31]. Similarly, Briassoulis and Giannoulis reported an EB of 33.8% for a corn starch and glycerol-based food film [32]. Significantly lower EB values of 5.12% and 4.18% were observed by Hou et al., 2020, for collagen films and collagen-chitosan blend films (in a 60–40 ratio), respectively [33]. A comparison with bio-based or commercial synthetic food films was not feasible, as these are produced through fully automated industrial processes and have thickness values two orders of magnitude lower than those obtained in our study. The results of mechanical testing suggest that the mucilage of *Ficus opuntia* and the silk fibroin solution do not significantly contribute to the improvement of the mechanical performance of the films. Therefore, they cannot be considered additives capable of improving the technological features of the film. This role is, however, unquestionably fulfilled by PVA and glycerol. The latter is present in all formulations and plays the crucial role of a plasticizer. It seems worth mentioning that chitosan films are rigid and need plasticizers to reduce the frictional forces between the polymer chains, such as hydrogen bonds or ionic forces, in order to improve mechanical performance. The incorporation of polyols in the formulation of the film can overcome this drawback [34].

### 3.2. Antioxidant Activity

**Figure 2:**
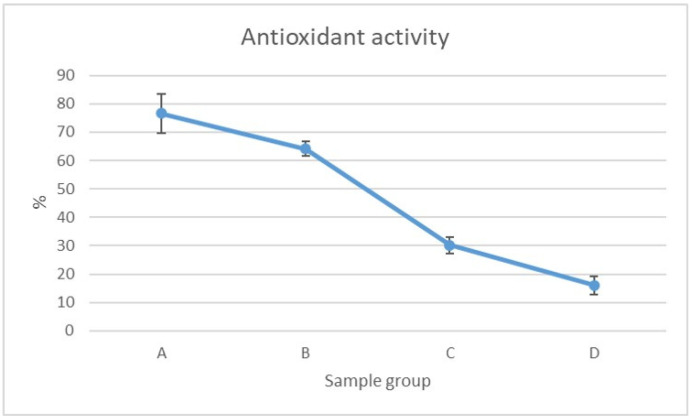
Radical scavenging activity of the four groups of chitofilms (see Section 2.3 for their description).

Figure 2 shows the antioxidant activity of the four chitofilms tested. The highest activity was observed for group A, namely the richest formulation that included PVA, glycerol, essential oils, Opuntia mucilage, and fibroin. The lowest values were registered for the group D samples, which were composed of only glycerol and chitosan. These findings support the addition of antioxidant agents to chitosan formulations in terms of the extension of shelf life. In fact, although the radical scavenging activity of chitosan is known and directly dependent on its hydrogen-donating ability, group D showed a very low antioxidant power. This suggests that the most significant contribution to the antioxidant activity of the film comes from constituents other than chitosan itself. This is the case of the mucilage from prickly pear, whose antioxidant properties are well documented and attributable to compounds such as vitamin E, carotenoids, phenols, flavonoids, betaxanthins, betacyanins, and amino acids [35]. Indeed, this natural bioactive was present in both the A and B groups, which reported the most intense antioxidant activity. The antioxidant power of chitosan and derivatives has been widely explored in the literature, sometimes demonstrated to depend strongly on its degree of polymerization and acetylation [24]. Moderate antioxidant activity was determined in a chitosan–eugenol complex nanoemulsion [36], while excellent values were observed in chitosan grafted with kogic acid [37] and with capsaicin [38].

### 3.3. Analysis of Wrapped Cheese

#### 3.3.1. Headspace

One way to assess the deterioration of processed cheese is by monitoring the development of volatile organic compounds (VOCs). Extensive research has been conducted on the volatile compounds in cheese, with microbial and light/water being identified as the cause of VOC formation during storage. Therefore, the analysis of the VOCs served as a means to evaluate how the type of wrapping (CTR and TRT) was correlated with the quality of preservation during storage. The composition of the headspace of cheese is sensitive to autooxidation and photooxidation, which might generate a wide array of VOCs, sometimes compromising the taste and appearance of the cheese.

**Figure 3:**
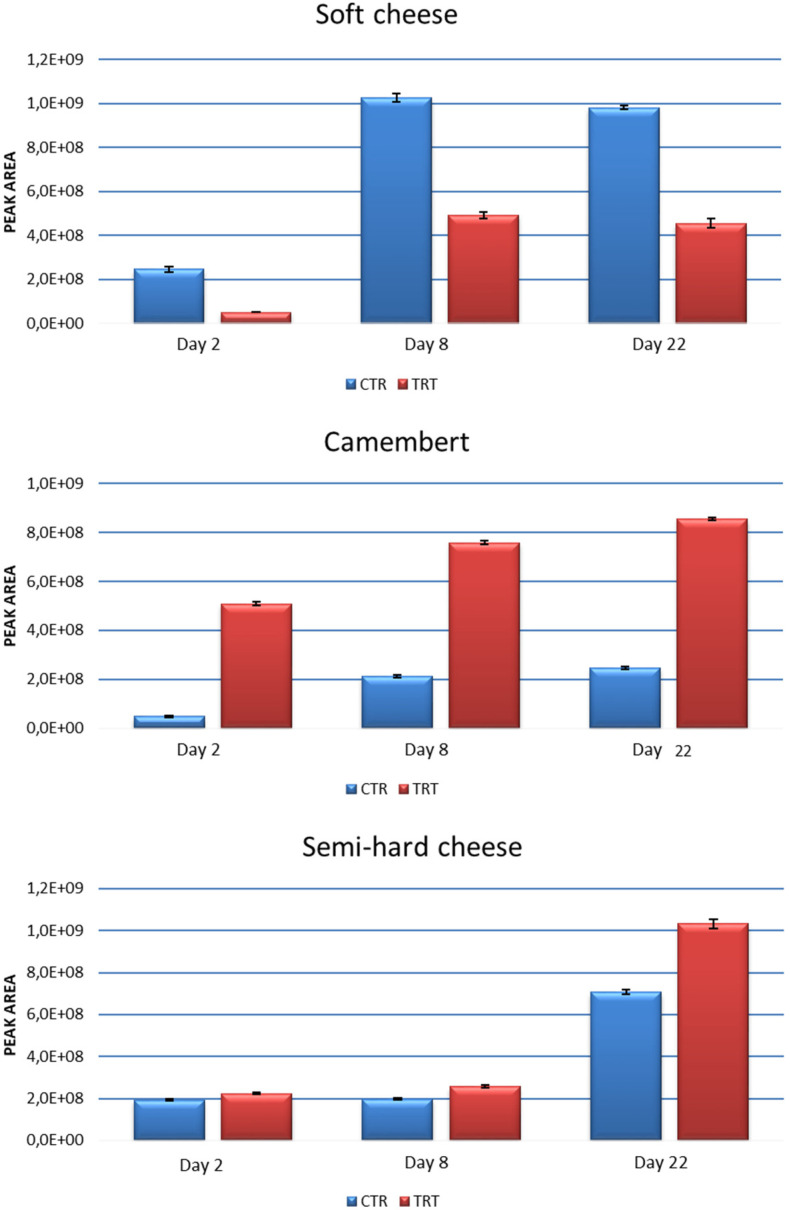
Contents (area counts) of the volatile organic compounds (VOCs) in the control (CTR) and treated (TRT) groups of cheese; SPME sampling occurred on days 2, 8, and 22 after cheese wrapping.

Preliminarily, a qualitative analysis of cheese headspace was carried out through SPME-GC-MS in order to choose the reference standards suitable for method validation. The six compounds reported in Table 2 are each representative of the main groups of VOCs found in cheese flavor. The LOQ, LOD, recovery, and range of linearity testify to the suitability of the developed SPME-GC method in terms of sensitivity, reliability, and accuracy [39]. Figure 3 shows the contents of VOCs as the GC area counts detected in the CTR and TRT groups of cheeses. With respect to soft cheese (top figure), the results point out that a more abundant volatile fraction developed in the CTR rather than in the TRT group. An opposite trend was observed for Camembert cheese, where the amount of volatiles becomes threefold in the TRT group.

**Figure 4:**
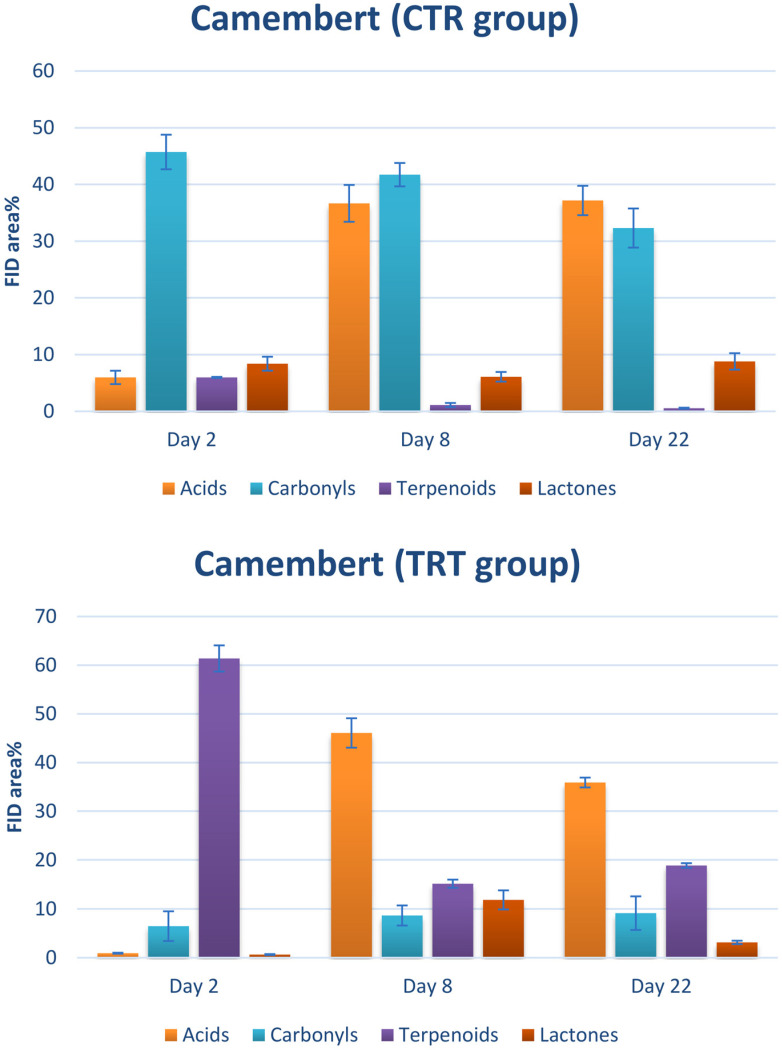
Classes of VOCs detected in the headspace of the Camembert cheese samples. CTR: control. TRT: treated.

This behavior can be widely justified by the analysis of single classes of analytes: as shown in Figure 4, the highest contribution in the TRT group was provided by terpenoids, characteristic components of essential oils [40]. In other words, this finding highlights a weak point in the chitofilm preparation procedure, particularly in the incorporation of the essential oil mix. It is evident that the mixture was not dispersed uniformly within the chitofilm matrix. On the other hand, no relevant differences could be caught between the CTR and TRT groups in semi-hard cheese (Figure 3, bottom). Nonetheless, in a group of samples (TRT, day 22), a tight sealing defect—the lack of adhesion of the film on the surface of cheese—was found, which was likely the cause of a marked increase of VOCs in these samples by triggering rancidity and oxidation phenomena.

**Figure 5:**
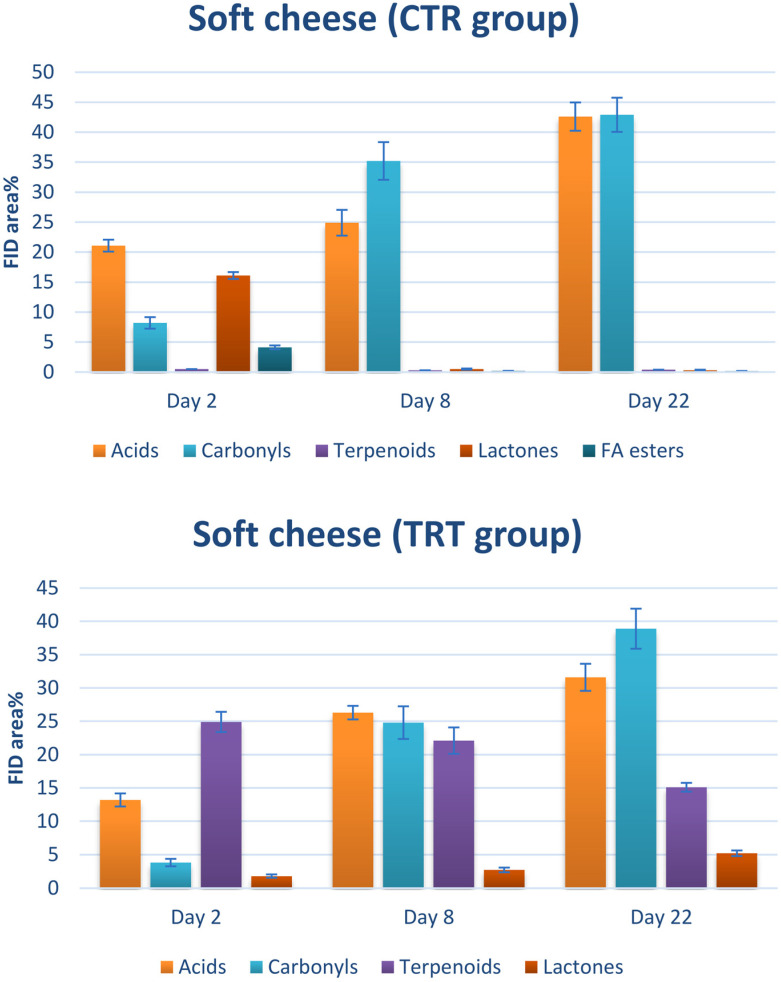
Classes of VOCs detected in the headspace of the soft cheese samples. CTR: control. TRT: treated.

**Figure 6:**
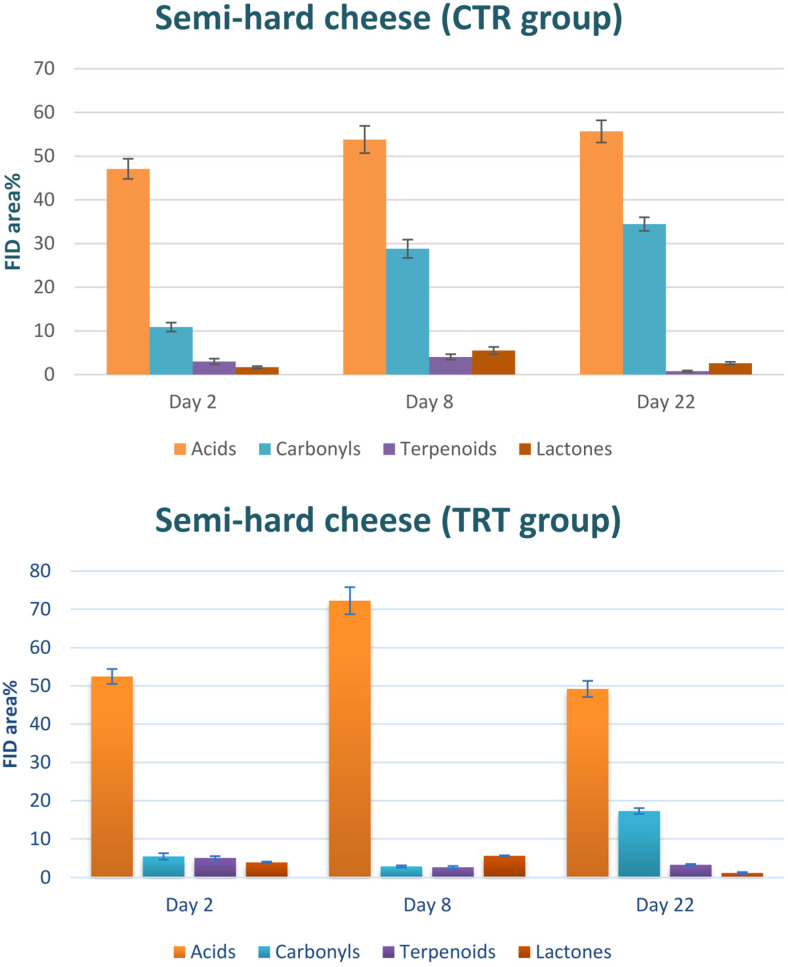
Classes of VOCs detected in the headspace of the semi-hard cheese samples. CTR: control. TRT: treated.

The volatile fingerprints were quite rich in peaks, with a chromatographic space becoming more crowded as the time of conservation became longer. This finding is in accord with previous works [41]. In order to make a fruitful comparison between the CTR and TRT samples, all the VOCs were grouped according to their chemical class. As can be seen in Figure 4, Figure 5 and Figure 6, the main classes contributing to the headspace composition were free fatty acids (i.e., butyric, capronic, caprylic, and capric), n-alkanals (i.e., hexanal, nonanal, and decanal), 2-alkenals (i.e., (2E)-hexenal, (2E)-heptenal, and (2E)-octenal), 2-ketones (i.e., 2-nonanone, 2-decanone, and 2-undecanone), lactones (i.e., γ-nonalactone, δ-decalactone, and γ-octalactone), and terpenoids (i.e., limonene, eugenol, thymol, and carvacrol). The minor compounds were esters and primary alcohols. All the assessed compositions are in accord with the literature with regard to the volatiles released by the cheese matrices [42].

Figure 5 illustrates that in the case of soft cheese, the rise in acids and lactones occurs promptly. However, after one week, there is a notable increase in aldehydes and ketones, and this pattern persists even through day 22. Within the TRT group, terpenoids consistently occupy approximately 15–25% of the chromatographic space, which aligns with expectations due to the addition of essential oils. In comparison to the other categories of the components, lactones, fatty acids, and carbonyls display a similar progression as observed in the CTR group.

Figure 4 depicts the distribution of volatile organic compounds (VOCs) observed in Camembert cheese. Notably, there is a significant presence of aldehydes and ketones detected as early as day 2 in the CTR group. This observation can be attributed to the specific characteristics of Camembert cheese, known for its delicate and subtle flavor, which is intimately linked to the milk used in its production. This milk carries the volatile compounds derived from the cattle’s pasture, particularly the fragrant grass. Indeed, among these constituents are terpenoids, which are known for their association with herbs and meadows. However, as the cheese continues to age, the proportion of fatty acids increases, accompanied by a rise in lactones. In the TRT group, aside from the previously mentioned issue related to essential oil addition, free fatty acids (FFAs) are more abundant than carbonyls. It can be hypothesized that the presence of essential oils may somewhat impede the degradation of casein by microbes, a well-known precursor of carbonyl compounds [43].

Figure 6 illustrates the composition of VOCs in semi-hard cheese. It is evident that FFAs play a dominant role in the volatile profile of both the CTR and TRT groups, aligning with previously documented information on the flavor constituents of matured cheese. In the CTR group, the distribution of VOCs remained consistent from day 2 to day 22. In contrast, the TRT group displayed a decline in carbonyls, mirroring the trend observed in Figure 4. Once again, it appears that the presence of essential oils may impede the formation of undesirable flavors.

In order to make a correct evaluation of the results presented, mentioning the mechanisms that preside over the formation of volatiles in cheese is useful. Microbial activity and the resulting breakdown of fats appear to play a significant role in shaping the primary components of the aroma in the majority of cheese varieties. The key aromatic elements in cheese, responsible for its flavor and scent, can be categorized as short-chain fatty acids and their derivatives, aldehydes, alcohols, ketones, and sulfur-based compounds. The degradation of amino acids is a pivotal process in the formation of a cheese flavor. Particularly, aromatic amino acids like phenylalanine, tyrosine, and tryptophan, as well as branched-chain amino acids such as leucine, isoleucine, and valine, alongside methionine, serve as the primary precursors for these aromatic compounds [43].

In general, as cheese ripens over time, the concentration of volatile components tends to increase. The predominant volatile compounds transition increases from five carbon atoms to a range of six to nine [44]. The microorganisms residing in the cheese play a crucial role in generating flavor compounds, particularly through the breakdown of lactate, protein degradation, fat decomposition, and lactose fermentation [45]. Casein, for instance, contributes to the production of amino acids, acetic acid, ammonia, pyruvate, aldehydes, alcohols, and carboxylic acids [43]. Lactones and citrate undergo catabolism, leading to the creation of lactate, diacetyl, acetoin, 2,3-butanediol, acetaldehyde, acetic acid, and ethanol. Butyric acid, hexanoic acid, and (2E)-nonenal play a significant role in creating the distinct aroma found in various types of cheese. The abundance of free fatty acids (FFAs) in all cheese varieties highlights the importance of lipolysis as a primary pathway for generating flavors.

Among the undesirable aromas, acetic acid stands out. In contrast to other acids, its origin is not connected to lipolysis; it results from the metabolism of citrate and lactic acid by lactic acid bacteria. The development of rancidity in cheese can be attributed to excessive or imbalanced lipolysis, leading to an overabundance of FFAs that produce undesirable aromas, such as γ-nonalactone. FFAs also serve as precursors to other potent aromatic compounds, including methylketones and esters. In this study, the wrapped cheese samples were intentionally kept in a refrigerator with a transparent door in order to simulate retail conditions. In fact, dairy products frequently face exposure to light during their storage and display in retail settings. It is well-established that light can trigger oxidation processes in food items, leading to the deterioration of valuable nutrients, changes in color, and the development of off-flavors, which often involve compounds like aldehydes and ketones.

Dairy products are particularly susceptible to light-induced reactions due to the presence of riboflavin (vitamin B2), a potent photosensitizer [46]. The process of autoxidation in unsaturated lipids entails the generation of free radicals, which subsequently leads to the formation of lipid oxidation products. This oxidation process results in the creation of various volatile carbonyl compounds, known for causing undesirable off-flavors. Previously, the assessment of the oxidative stability of cream cheese stored in transparent and white trays was conducted through the quantification of volatiles as the area percentage [46]. In summary, the presence of light substantially increases the concentrations of aldehydes (i.e., hexanal), while exposure to higher temperatures is positively associated with increased levels of 2-ketones, octanal, nonanal, and decanal. Conversely, rising temperatures lead to a decrease in acetoin and its oxidized form, known as 2,3-butanedione.

#### 3.3.2. Microbiology

Table 3 shows the trend of bacterial growth for mesophilic (MES), enterobacteriaceae (ENT), and lactic acid (LAC) in both the CTR and TRT groups. Internally to the two investigated groups, the results, which are expressed as the mean of the colony forming unit (CFU)/mL for each type of cheese, were collected according to the time of the microbiological evaluation, namely day 2, day 8, and day 22. In general, the TRT samples demonstrated an inhibitory activity on the bacterial growth that was either comparable or lower than those observed in the CTR samples. In soft cheese, at day 2, the mesophilic bacteria counts in the TRT group were an order of magnitude lower than those detected in the CTR group (2.9 × 10^2^ CFU mL^−1^ vs. 1.3 × 10^3^ CFU mL^−1^). However, this trend was inverted during storage. The bacterial growth of enterobacteriaceae and lactic acid bacteria was consistently comparable in the CRT and TRT samples of soft cheese, Camembert, and semi-hard cheese. The antimicrobial properties of polyethylene food films are undoubtedly associated with the characteristics of their components, particularly the presence of synthetic chemical additives capable of exerting the action of “food preservatives”.

Among these, the most relevant are silica and titanium dioxide nanoparticles [47], ethanol, chlorine dioxide, and sulfur dioxide [48]. In chitofilms, the antimicrobial action relies entirely on the combined effects of its natural constituents, namely chitosan, essential oils, nopal, and silk fibroin. The comparable levels of bacterial growth in the TRT samples to those obtained in the CTR group confirm that the use of natural substances with antimicrobial properties is a valid and sustainable alternative in the production of active food wraps. The most significant action is undoubtedly exerted by essential oils, which easily penetrate the bacterial cell wall because of the hydrophobic nature and lipophilic character of their components, interfering with molecular transport mechanisms and leading to cellular inactivation. The results affirm the effectiveness of essential oils in inhibiting the growth of Gram-positive and lactic acid bacteria, the latter typical of cheese metabolism. 

The hydrophobic nature of certain volatile fraction components allows them to penetrate the cell wall, primarily composed of peptidoglycans, teichoic acid, and proteins [49]. In particular, the phenyl compounds present in essential oils exhibit the highest inhibitory action against Gram-positive bacteria, successfully interfering with the enzymes involved in energy production and denaturing proteins [50]. Some of these compounds, such as thymol, carvacrol, camphor, and p-cymene, have been found in significant quantities in the essential oils of aromatic herbs used in the chitosan film (see Section 3.3.1). The presence of phenolic compounds in nopal further contributes to enhancing this effect [51]. Similarly, the results confirm the presence of phenylpropanoids (i.e., eugenol and chavicol), which exhibit inhibitory action against Gram-negative bacteria such as enterobacteriaceae.

The antimicrobial activity of eugenol is particularly linked to the presence of the double bond in the alpha and beta positions of the side chain and the methyl group in the gamma position. The choice to use a blend of essential oils from aromatic herbs is therefore valid, as the presence of different compounds allows for effective action against both Gram-positive and Gram-negative bacteria, enhancing the antimicrobial character of the chitosan film [50]. However, in the specific case of cheeses, it can be presumed that the antimicrobial nature of the film is moderately inhibited, as the fats present in cheeses may encapsulate the hydrophobic constituents of essential oils, limiting their ability to access the target sites of microorganisms. Nevertheless, this effect is mediated by the lower pH, which enhances the hydrophobicity of essential oils, thereby improving their ability to attack the cell wall [49]. The literature on the antimicrobial activity of chitosan and congeners is abundant; several of the studies that present chitosan-based formulations tested for their biocompatibility, including their capability to kill microbes [52,53,54].

#### 3.3.3. Sensory Analysis

**Figure 7:**
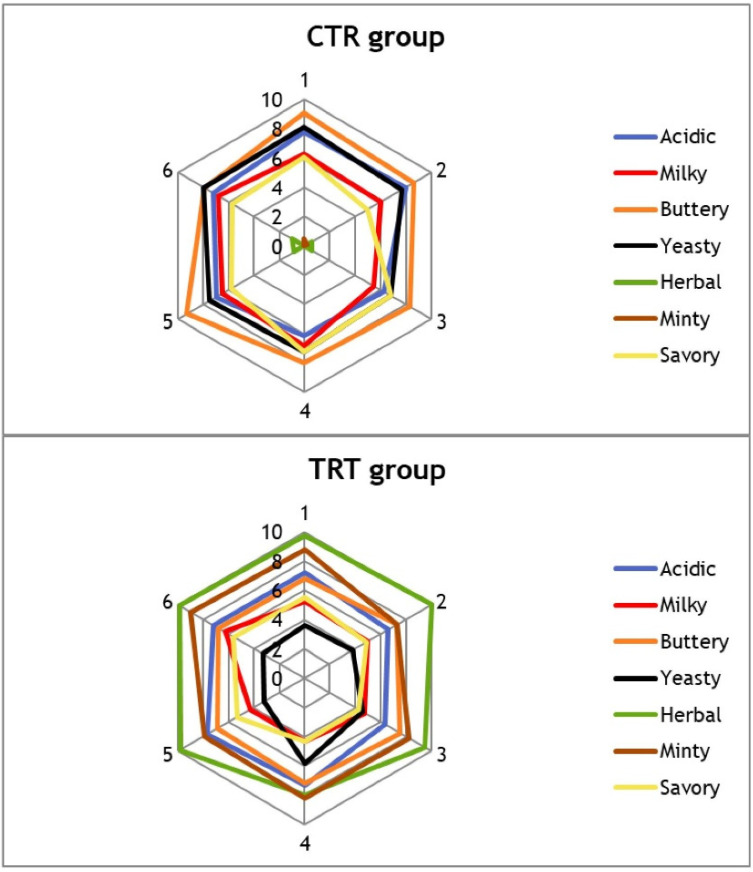
Spider diagrams showing the sensorial attributes of the cheese samples perceived by the panelists. CTR: control. TRT: treated.

As depicted in Figure 7, the panel observed notable distinctions between the two cheese groups. All attributes were discernible except for “minty” and “herbal”, which were not detected in the CTR group. The latter exhibited higher scores for attributes such as yeasty (7.6 vs. 4.1), milky (6.3 vs. 5.0), buttery (8.5 vs. 7.1), and savory (6.1 vs. 5.0), while both groups received the same rating for the acidic attribute (6.1 vs. 6.1). This preliminary descriptive analysis of the sensory characteristics of the cheese samples served as an initial means to determine if the composition of the film could impact the cheese taste. In other words, the sensory analysis aimed to assess whether any ingredient migration from the film to the cheese matrix might compromise the flavor and appearance of the food. The results clearly demonstrated that: (i) the inclusion of the essential oil blend significantly altered the taste of the cheese stored in the chitofilm, primarily due to its strong odor impact; and (ii) the choice of essential oils, useful for prolonging the cheese shelf life, must be made carefully, as their aroma should harmonize with the food they come into contact with. Not so numerous are the papers that explore the sensory properties of foods wrapped in chitosan-based packaging. Song et al. investigated the effects of glycerol-plasticized chitosan on the freshness of ground meat, and the results were compared to PE films, confirming that the CS film procrastinated the loss of freshness more than the PE film [55]. Another work applied a sensory panel to the evaluation of odor and color of cheese wrapped in bio-based and conventional PVC films, evidencing a higher tendency for color variation and odor decay over time in bio-based vs. PVC cling films [56].

## 4. Conclusions

In conclusion, this study represents a significant advancement in chitosan-based packaging technology by incorporating specialized ingredients, such as PVA, silk proteins, and *Ficus opuntia* mucilage, never reported before in the formulation of a chitosan-based cling film. In fact, the inclusion, among others, of Opuntia shovel mucilage strongly enhances the antioxidant power (from 16% to 76%) of the cling film, thus making it a valid tool for ameliorating the stability of food shelf life. The mechanical features and the performance as a type of wrapping packaging of the novel film were tested; the tensile strength ranged from 12.10 to 23.25 MPa, whereas the elongation at break ranged from 27.91% to 46.12%.

Three cheese varieties were chosen as food models to be wrapped in the chitofilm. Volatiles entrapped into the headspace of the film were monitored through SPME-GC-MS during storage. The overall volatile fingerprints became richer over time, in accordance with previous studies. The incorporation of essential oils in the formulation appeared as a critical issue due to the lack of uniformity and homogeneous dispersion in some cases. These findings were confirmed by a sensory analysis that revealed notable distinctions between the CTR and TRT cheese groups. Quali-quantitative differences in the composition of the volatile fraction were specifically linked to each sample group, although a general trend could be observed in the TRT group, where a potential inhibitory effect of the essential oils on the formation of undesirable flavors was observed.

Mesophilic, enterobacteriaceae, and lactic acid bacteria were assayed at three different times for each type of cheese: the highest load was quantified as 8.2 × 10^6^ CFU/mL at day 22 in the CTR Camembert cheese. The results obtained in this study encourage the transfer of chitofilm production from the laboratory to an industrial scale by preliminarily solving some minor technical issues observed during the experimental preparation of the four proposed formulations. Further research in this direction may contribute to refining the film formulation presented here, which widely falls within the strategies for the sustainable development of the global community. It is important to reiterate that in this study, the chitosan extraction occurred through the fermentation of wasted shellfish, unlike the much more common methodology based on the use of large quantities of chemicals with a high environmental impact.

## 5. Patents

The patent entitled “Edible bio-packaging”, deposited in 2021 with the number 102021000013913 and issued by the Italian Ministero delle Imprese e del Made in Italy on 11 July 2023. 

## Figures and Tables

**Table 1:** Parameters evaluated in the mechanical testing of different film formulations (see text for descriptions). FT: film thickness; TS: tensile strength; EB: elongation at break.

Sample Group	FT [µm]	TS [MPa]	EB [%]	Hardness [Shore A]
A Mean ± SD	309.00 ^ns^ ± 0.81	23.25 ^a^ ± 0.48	46.12 ^a^ ± 7.94	71 ^a^ ± 1.11
B Mean ± SD	308.21 ^ns^ ± 1.12	19.44 ^b^ ± 0.64	38.60 ^b^ ± 4.11	55 ^b^ ± 1.25
C Mean ± SD	309.11 ^ns^ ± 0.94	13.63 ^c^ ± 0.56	34.35 ^c^ ± 0.99	52 ^b^ ± 1.13
D Mean ± SD	308.57 ^ns^ ± 0.68	12.10 ^d^ ± 0.81	27.91 ^c^ ± 1.39	54 ^b^ ± 2.28

ns: no significant difference (*p* ≥ 0.05). Different superscript letters in the same row indicate significantly different values (*p* < 0.05 using Tukey’s post hoc HSD test); the same superscript letters in the same column indicate not significantly different values (*p* > 0.05 using Tukey’s post hoc HSD test).

**Table 2:** Validation data of the SPME-GC method. LOQ: limit of quantification; LOD: limit of detection.

Compound	LOQ (ng·g^−1^)	LOD (ng·g^−1^)	% Recovery (%RSD)	Range of Linearity (mg·g^−1^)
n-Hexanal	1.10	0.31	96.3 (8.4)	0.1–10.0
Octanoic acid	2.42	0.85	102.7 (14.3)	0.1–10.0
Ethyl tetradecanoate	0.48	0.09	85.7 (7.9)	0.1–10.0
δ-Decalactone	0.31	0.12	98.7 (8.1)	0.1–10.0
Limonene	0.42	0.26	97.4 (10.9)	0.1–10.0
Dodecane	0.53	0.27	97.9 (9.4)	0.1–10.0

**Table 3:** Trend of bacterial growth observed in the different types of cheese investigated during shelf life.

Group	Sampling	Sample Type	MES	ENT	LAC
Control	Day 2	Soft cheese	1.3 × 10^3^	n.d.	8.4 × 10
		Camembert	4.6 × 10^4^	n.d.	9.4 × 10^3^
		Semi-hard	1.1 × 10^5^	0.5 × 10	5.7 × 10^4^
	Day 8	Soft cheese	1.2 × 10^2^	n.d.	5.0 × 10^5^
		Camembert	6.1 × 10^3^	n.d.	6.7 × 10^5^
		Semi-hard	1.9 × 10^3^	n.d.	4.5 × 10^4^
	Day 22	Soft cheese	6.2 × 10^5^	n.d.	1.1 × 10^4^
		Camembert	8.2 × 10^6^	9.9 × 10^2^	8.0 × 10^5^
		Semi-hard	1.9 × 10^6^	n.d.	1.2 × 10^4^
Treated	Day 2	Soft cheese	2.9 × 10^2^	0.5 × 10	7.0 × 10
		Camembert	9.2 × 10^4^	0.5 × 10	8.2 × 10^3^
		Semi-hard	1.3 × 10^5^	0.5 × 10	1.1 × 10^5^
	Day 8	Soft cheese	6.1 × 10^3^	n.d.	4.4 × 10^5^
		Camembert	1.4 × 10^3^	n.d.	2.5 × 10^5^
		Semi-hard	1.7 × 10^3^	n.d.	7.8 × 10^4^
	Day 22	Soft cheese	2.2 × 10^6^	n.d.	3.2 × 10^4^
		Camembert	1.3 × 10^6^	0.3 × 10	6.7 × 10^5^
		Semi-hard	1.1 × 10^6^	n.d.	1.6 × 10^5^

MES: mesophilic; ENT: Enterobacteriaceae; LAC: lactic acid bacteria; n.d.: not detected.

## Data Availability

The original contributions presented in the study are included in the article/Supplementary Materials, further inquiries can be directed to the corresponding author.

## Supplementary Materials

The following supporting information can be downloaded at: https://www.mdpi.com/article/10.3390/foods13132055/s1, Figure S1: Physical properties of four different film formulations. WVP: water vapor permeability; LT: light transmission; MC: moisture content; WS: water solubility. Figure S2: Testing of “elongation at break” of the four different chitofilms. Figure S3: Cheese samples wrapped in the chitofilm. 1: Camembert cheese; 2: semi-hard cheese; 3: soft cheese. Figure S4: Headspace-SPME-GC chromatograms of Camembert cheese samples (CTR group). A: sampling day, 2; B: sampling day, 8; C: sampling day, 22. Figure S5: Evaluation form administered to panelists during the sensory analysis. Figure S6: Macroscopic image of the chitosan-based film (Group A) conditioned on a plexiglass plate. Figure S7: Representative SEM images of group A, B, C, D cling films. Scale bar 30 µN and magnification of 200 x at 20 kV. Table S1: indicators of freshness and quality of the cheeses used for the experimental activity. Table S2: (a) Mechanical properties of the PE film; (b) barrier properties of the PE film.
