# Supplementary material for: Development of a Chitosan-Based Film from Shellfish Waste for the Preservation of Various Cheese Types during Storage
Source: Foods. 2024 Jun 27;13(13):2055. doi: 10.3390/foods13132055 (PMC11241246; doi:10.3390/foods13132055)
Supplement: Supplementary file 1 [file foods-13-02055-s001.zip › Suppplementary figures.pdf]

Supplementary figures

Figure S1: Physical properties of four different film formulations. WVP: water vapor permeability; LT: light transmission; MC: moisture content; WS: water solubility.

Figure S2: Testing of "elongation at break" of the four different biofilm formulations.

Figure S3: Cheese samples wrapped in the chitofilm. 1: camembert cheese; 2: semi-hard cheese; 3: soft cheese.

Figure S4: Headspace-SPME-GC chromatograms of Camembert cheese samples (CTR group). **A**: sampling day, 2; **B**: sampling day, 8; **C**: sampling day, 22.
