# Supplementary figures and images for: Development of a Chitosan-Based Film from Shellfish Waste for the Preservation of Various Cheese Types during Storage
Source: Foods. 2024 Jun 27;13(13):2055. doi: 10.3390/foods13132055 (PMC11241246; doi:10.3390/foods13132055)

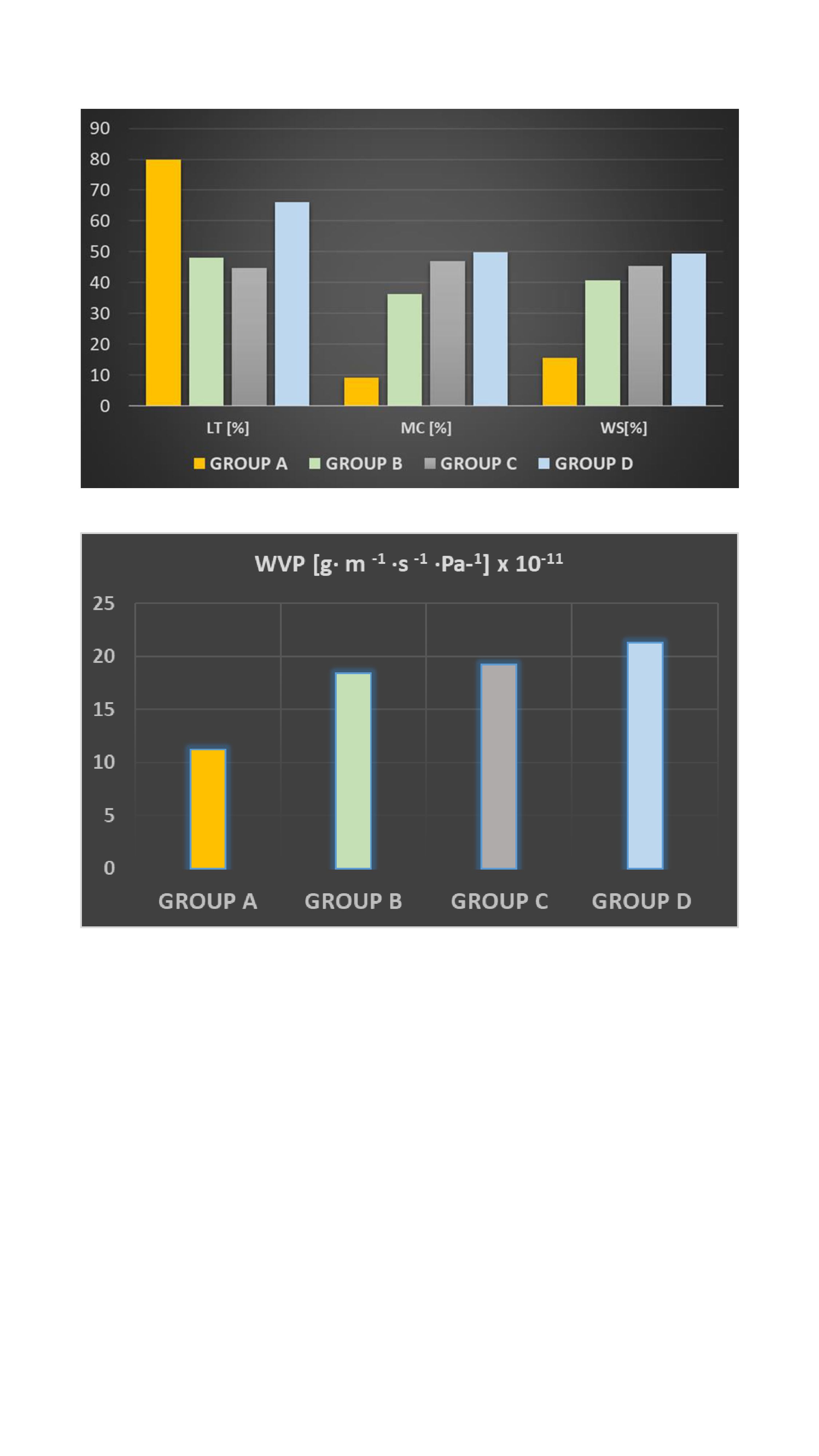

Supplement: Supplementary file 1 [file foods-13-02055-s001.zip › Supplementary Materials/Figure S1.jpg]

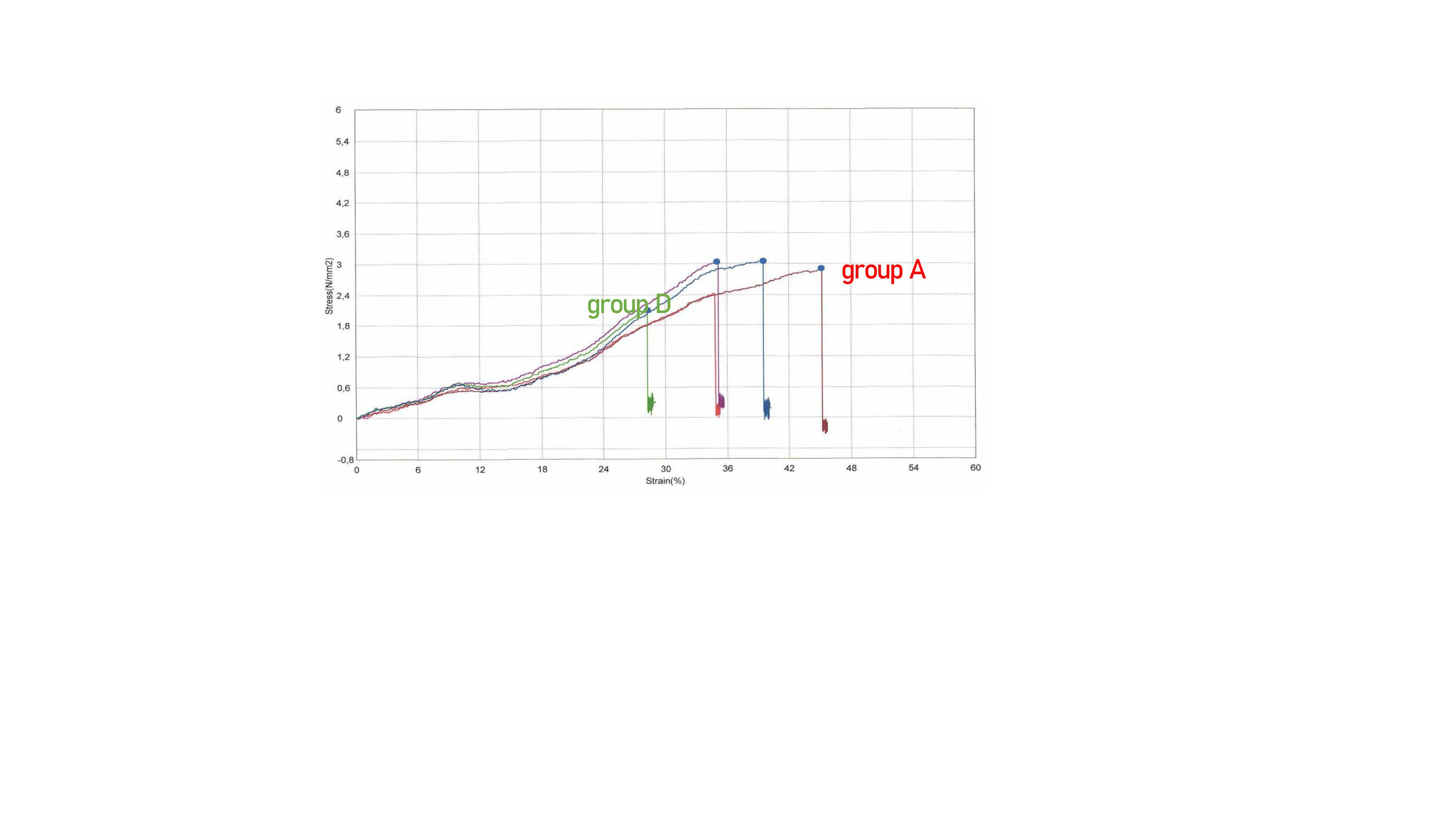

Supplement: Supplementary file 1 [file foods-13-02055-s001.zip › Supplementary Materials/Figure S2.jpg]

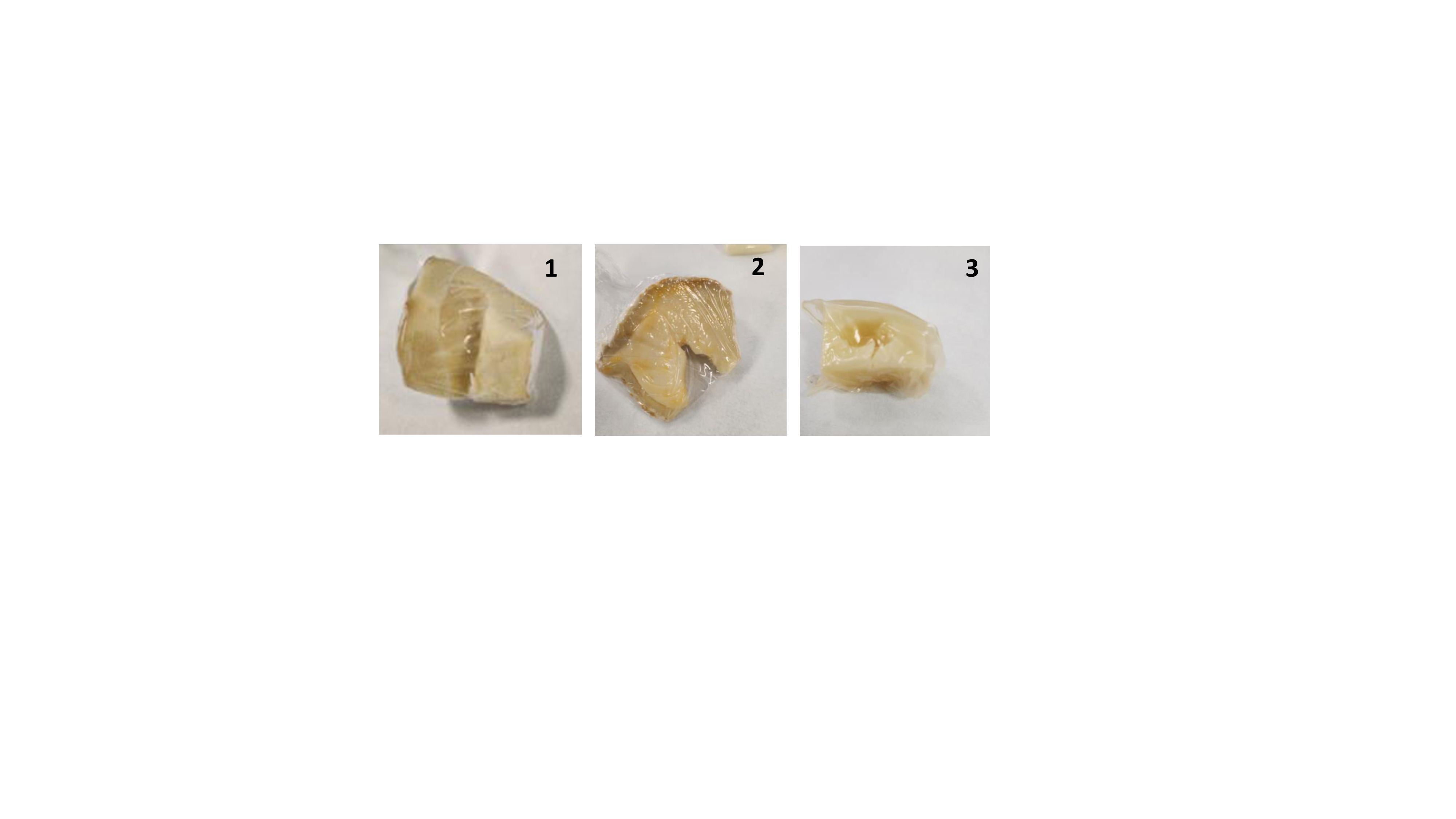

Supplement: Supplementary file 1 [file foods-13-02055-s001.zip › Supplementary Materials/Figure S3.jpg]

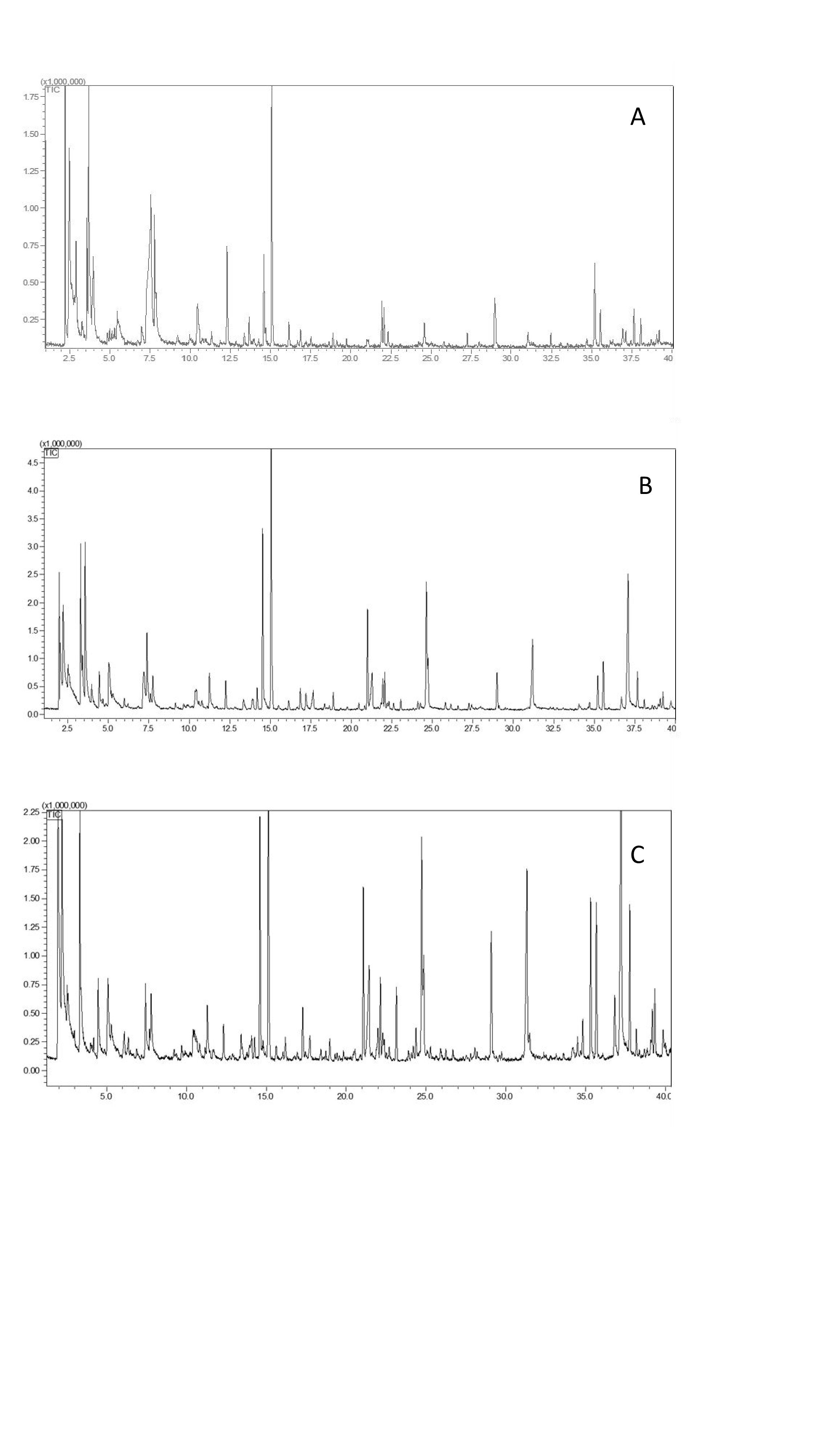

Supplement: Supplementary file 1 [file foods-13-02055-s001.zip › Supplementary Materials/Figure S4.jpg]
